# Orphan and *de novo* Genes in Fungi and Animals: Identification, Origins and Functions

**DOI:** 10.1093/gbe/evaf220

**Published:** 2025-11-25

**Authors:** Ercan Seçkin, Dominique Colinet, Edoardo Sarti, Etienne G J Danchin

**Affiliations:** Institut Sophia Agrobiotech, INRAE, Université Côte d’Azur, CNRS, Sophia-Antipolis 06903, France; Algorithms, Biology Structure, Centre Inria at Université Côte d’Azur, Sophia-Antipolis 06902, France; Institut Sophia Agrobiotech, INRAE, Université Côte d’Azur, CNRS, Sophia-Antipolis 06903, France; Algorithms, Biology Structure, Centre Inria at Université Côte d’Azur, Sophia-Antipolis 06902, France; Institut Sophia Agrobiotech, INRAE, Université Côte d’Azur, CNRS, Sophia-Antipolis 06903, France

**Keywords:** evolution, orphan genes, *de novo* gene birth

## Abstract

Genes that lack identifiable homologs in other species have been an intriguing and interesting topic of research for many years. These so-called orphan genes were first studied in yeast and since then, they have been found in many other species. This has fostered a whole field of research aiming at tracing back their evolutionary origin and functional significance. Orphan genes represent an important part of protein-coding genes in many species. Their presence was initially mainly hypothesized to result from high divergence from a pre-existing gene, with duplications or horizontal gene transfer facilitating their accelerated evolution. More recently, their possible *de novo* emergence from nongenic regions has gained particular interest. Several orphan genes are predicted to be involved in reproduction, while others are involved in specific developmental stages, in adaptation mechanisms such as freeze protection or even human disease. However, there is currently no unified resource or synthesis that brings together existing knowledge about how prevalent orphan genes are across different species and what their roles might be. In this review, we focus on orphan genes in animals and fungi. We provide a detailed summary of discoveries over time in terms of orphan gene prevalence in genomes, their origins as well as their roles in different biological contexts.

Significance Orphan and *de novo* genes are found in all lineages and often contribute to lineage- or species-specific adaptations, yet their origins and functions remain poorly understood. While multiple case studies exist, a recent unified synthesis focusing on their prevalence, biological roles, and detection methods across animals and fungi is timely. This review brings together current knowledge from both model and nonmodel species, comparing how orphans, including *de novo* genes are identified, and highlights methodological challenges that affect their classification as well as their possible functions. Our work provides a foundation for future studies aiming to clarify their evolutionary significance and potential applications in health, agriculture, and biodiversity research.

## Introduction

### Orphan Genes and *de novo* Gene Birth

The definition of orphans varies across fields of research: some use the term for genes of unknown function (Hartig et al. 2011), whereas others refer to receptors that lack known ligands (Nothacker 2008), regardless of whether or not these so-called orphans have homologs in other species. Here, we use the more classical evolutionary biology definition, which refers to orphans as genus- or lineage-specific genes lacking homologs in other species. It is important to note that being orphan depends on the focal phylogenetic branch which varies from study to study and is commonly referred to as phylo-stratygraphy. Orphan genes have been first described in the *Saccharomyces cerevisiae* yeast genome (Dujon 1996) and were initially predicted to represent up to 30% of protein-coding genes in eukaryotes (Tautz and Domazet-Lošo 2011), even though this percentage will be discussed later on in the light of other studies. Their emergence represents an important opportunity for the acquisition of new functions during evolution, in particular by driving genus or species-specific adaptations (Fakhar et al. 2023). Orphan genes may derive from pre-existing ones that have diverged in their sequence to the point of no recognition of homology (Tautz and Domazet-Lošo 2011). This can be facilitated by gene duplication or horizontal gene transfer events, followed by rapid evolution. Studies in multiple species suggest, however, that this explanation concerns only a part of existing orphan genes (Vakirlis et al. 2020a). Another, not mutually exclusive, hypothesis is that orphan genes may emerge from nongenic regions. This phenomenon, known as *de novo* gene birth, occurs when previously noncoding and/or not transcribed DNA sequences acquire the capacity to be both transcribed and translated to a functional protein (Schmitz and Bornberg-Bauer 2017; Weisman 2022). For a long time, *de novo* emergence was considered unlikely as the probability of a new gene coding for a functional protein being maintained in populations by selection is low (Jacob 1977; Schlötterer 2015). With the increase in available and high-quality genomic data, it was realized that *de novo* gene emergence is not as rare as initially thought (Vakirlis et al. 2020a). Several studies took advantage of a richer set of genome data to confirm the likely existence of *de novo* emerged genes (Tautz and Domazet-Lošo 2011; McLysaght and Hurst 2016; Van Oss and Carvunis 2019). The most recent and comprehensive review illustrating the importance of *de novo* emerged genes was published in 2024 and it provides detailed information, including the methods to identify them, their possible functions and the challenge they still pose from an evolutionary biology point of view (Zhao et al. 2024a). As a rapidly evolving field of research, several further studies have recently brought clarifications on different open questions.

The functions of the majority of orphan genes are still unknown, as most lack known motifs, domains, recognizable folds, or reliable protein structure predictions (Fakhar et al. 2023). However, there has been huge progress in the field, providing several clues to the functions of orphan genes in different species (Fakhar et al. 2023). These progresses have mainly been achieved by combining biochemical and experimental structure analysis, and also by studying the expression patterns of orphan genes, including *de novo* genes, in different compartments of an organism.

### Mechanisms of *de novo* Gene Birth

**Fig. 1.**
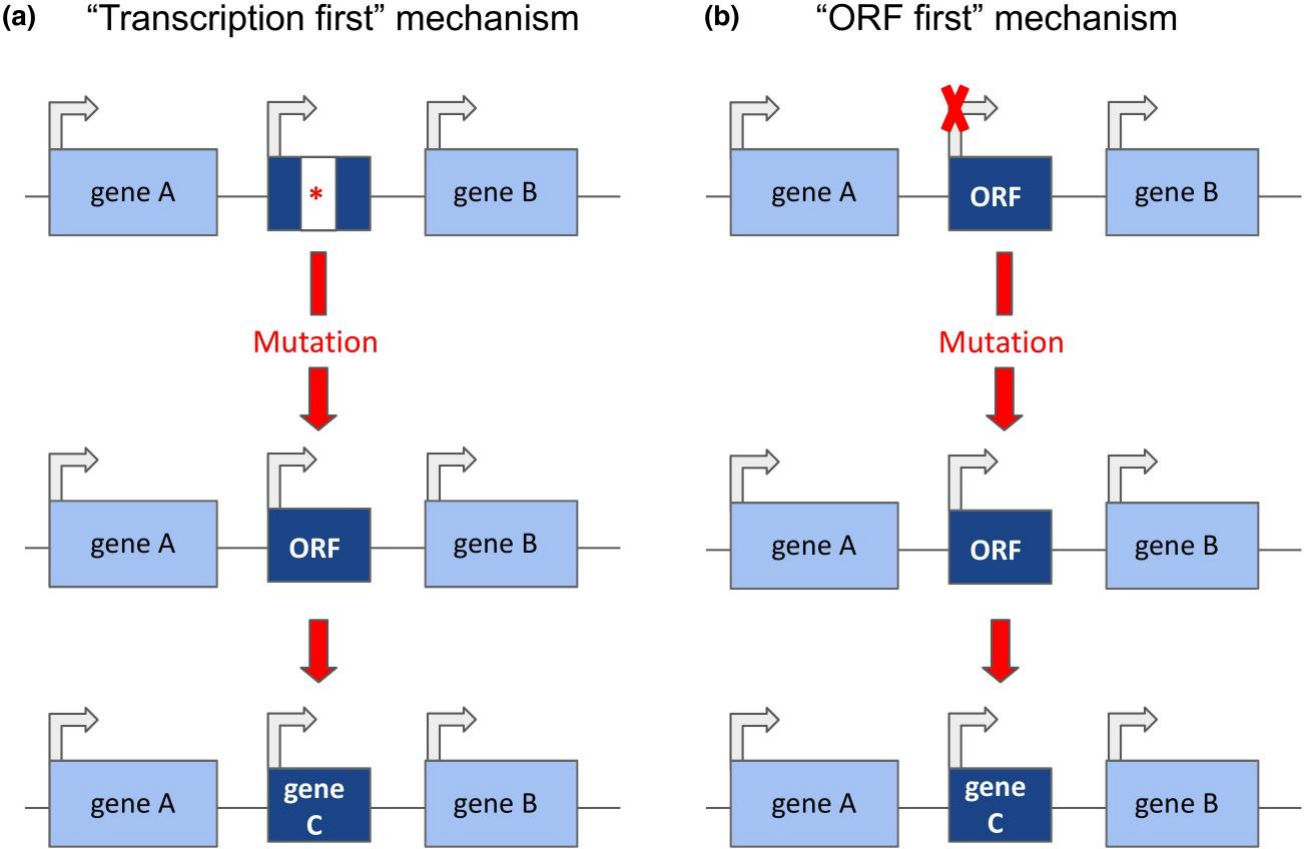
Emergence of a *de novo* protein-coding gene from a nongenic region. Gene C represents the *de novo* gene resulting from the mechanisms described. a) In the “transcription first” mechanism, a nongenic sequence mutates resulting in premature stop codons (asterisk in the middle) being removed, yielding an ORF and a new gene. b) In the “ORF first” mechanism, acquiring an expression regulatory region (gray arrows) allows transcription of a pre-existing ORF and yields a new gene.

In the case of a protein-coding gene, *de novo* emergence is hypothesized to involve two main distinct processes: (i) transcription of initially noncoding DNA and (ii) acquisition of an open reading frame (ORF) (Fig. 1). The order of these events allows two main mechanisms to be distinguished (Van Oss and Carvunis 2019): “transcription first” (Fig. 1a) and “ORF first” (Fig. 1b).

Although still debated, the “transcription first” model is thought to be prevalent because many nongenic sequences are transcribed (Van Oss and Carvunis 2019; Grandchamp et al. 2024), which is commonly referred to as pervasive transcription and mathematical models suggest that under neutral evolution transcription first is more likely (Iyengar and Bornberg-Bauer 2023). These sequences usually lack a canonical ORF due to a missing start codon, or premature stop codons, frameshifts and/or nonfunctional splice sites. The accumulation of mutations can result in the emergence of an ORF, and consequently, a *de novo* coding gene that can be translated into a protein. Such intermediate sequences are named protogenes (Carvunis et al. 2012). Protogenes may initially produce weak or harmful proteins or peptides, and many are eliminated by natural selection. In rare cases, however, a protogene can provide a slight benefit to the organism, leading to its retention and refinement through beneficial mutations. Over time, this suite of events is hypothesized to result in the fixation of the protogene and its evolution into a fully functional gene.

The “ORF first” mechanism involves an ORF that is present in the genome but not transcribed due to the absence of a regulatory region controlling its expression. Mutations accumulating in this region can yield a promoter or regulatory sequence, allowing the ORF to be transcribed and giving rise to a *de novo* gene. *De novo* transcription of a genomic region can be facilitated by the insertion of a transposable element and its regulatory regions near an ORF (Lebherz et al. 2024).

However, it is important to note that the distinction between “transcription first” and “ORF first” mechanisms is not always straightforward. Just as it can be difficult to definitively classify an orphan gene as *de novo* or highly diverged, the temporal sequence of transcription and ORF acquisition may not be neatly separated. For example, an ORF formed in a region of low transcription may gradually acquire regulatory features, or a *de novo* gene may later undergo rapid divergence that obscures its origin (Prabh and Rödelsperger 2019).

### Methods to Identify Orphan Genes and *de novo* Gene Birth

The most common approach to identify orphan genes is to start from a focal branch in the tree of life and search for homologs in other species using comparative genomics. One of the most widely used methods is phylostratigraphy, which involves identifying homologs for all genes from a species or clade of interest in the rest of the species using homology detection methods. It should be noted here that most of these methods use protein sequences as a proxy for protein-coding genes, due to their higher conservation. Then, based on these searches, groups or clusters of homologous genes are built using state-of-the-art software such as OrthoFinder (Emms and Kelly 2019), ORFan-Finder (Ekstrom and Yin 2016), GenEra (Barrera-Redondo et al. 2023) or SonicParanoid (Cosentino and Iwasaki 2023). The identification of a gene exclusively within one or few closely related species enables the determination of the probable relative date of gene emergence, as well as the classification of the gene as orphan. The differences between orphan gene identification methods using comparative genomics have already been examined in detail in another review (Fakhar et al. 2023).

From an initial dataset of orphan genes, *de novo* genes can be identified by aligning the corresponding proteins to the genome of a closely related species translated in its 6 frames and looking for similarities in the corresponding noncoding regions (Vakirlis et al. 2020a). If the corresponding region in the related species is noncoding and mutations can be identified at specific positions that have led to the acquisition of an ORF, a *de novo* emergence event can be assumed. However, in case of high divergence, establishing reliable correspondences between genomes can be difficult. Translocations, structural changes, or incomplete assemblies can also obscure the ancestral origin of a gene (Vakirlis et al. 2020a). Distinguishing between *de novo* genes and highly diverged homologs is particularly challenging because highly diverged homologs no longer have detectable sequence similarity, making them appear to have arisen “from scratch”. Conversely, *de novo* genes arise from noncoding sequences that may superficially resemble highly divergent homologs, further complicating their identification (Vakirlis et al. 2020a; Weisman et al. 2020).

Nevertheless, incorporating the broader genomic context via conserved synteny analysis helps disentangle between these two possibilities (Zhao et al. 2014; Vakirlis et al. 2020a). This consists in determining whether genes surrounding the candidate orphan gene in the focal species are in conserved synteny or collinear in target closely related species. In case of synteny conservation of the surrounding genes, then the next step is to examine the homologous target locus corresponding to the candidate orphan gene. If at this target locus, another gene is present but lacks homology to the orphan gene, then we can hypothesize the orphan gene has highly diverged from a common ancestral gene. Conversely, if at this locus there is no predicted gene but partial alignment of the orphan gene with frameshifts and/or invalid splice sites, then the *de novo* gene birth hypothesis is more likely (Ruiz-Orera et al. 2015; Vakirlis et al. 2020a).

In recent years, new tools have been developed to facilitate the study of orphan and *de novo* genes by integrating existing methods into streamlined pipelines. One such tool is DENSE (Roginski et al. 2024), which combines comparative genomics, synteny analysis and expression data to identify candidate *de novo* genes. While such tools represent an important step towards standardizing and simplifying *de novo* gene discovery, they are not yet widely used, as they remain very recent. Methodology for the identification of *de novo* genes is more broadly discussed in a recent review (Grandchamp et al. 2025).

## Orphan Genes Identified and Functionally Studied in Fungi and Animals

**Fig. 2.**
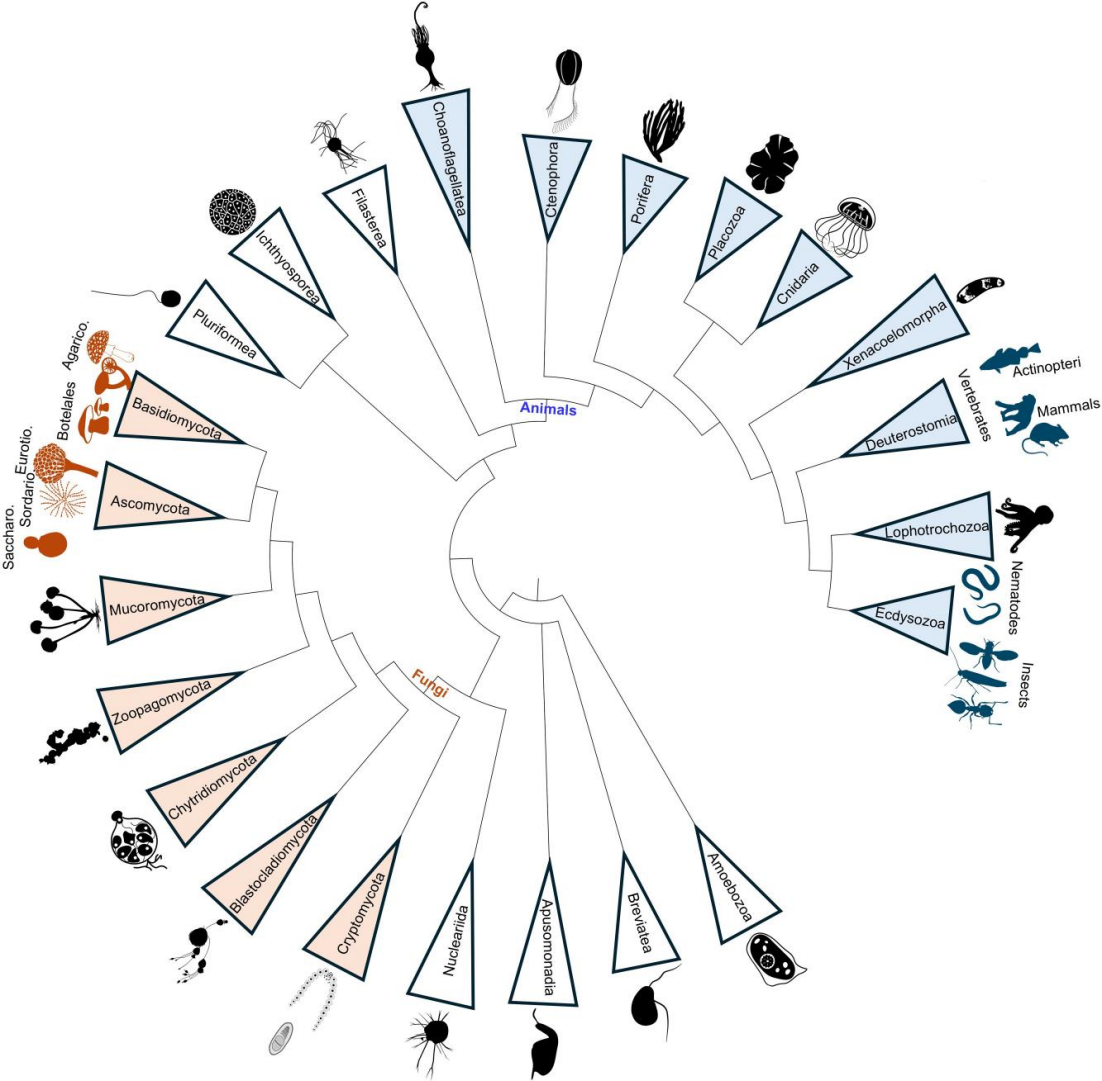
Schematic phylogeny of opisthokonts highlighting fungal and animal species in which orphan genes have been studied. The tree topology has been extracted from a recent phylogenomics analysis of opisthokonts (Liu et al. 2024). Phylogenetic groups corresponding to animals are colored in blue while those corresponding to fungi are colored in orange. Illustrative silhouettes of the different groups have been retrieved from PhyloPic (https://www.phylopic.org/). Colored silhouettes highlight species that have been studied while those that are unexplored have been left in dark.

Since, historically, orphan genes were first described in yeast, we first reviewed orphan gene cases in yeasts, then besides yeast in other fungi and finally more broadly in other opisthokonts such as animals, including humans (Fig. 2). Therefore, in the following sections, we will review the most prominent cases of highly divergent and *de novo* orphan genes by phylogenetic groups in chronological order to show how much these genes contribute to the genomes of the studied species, how they are identified, and what has changed over time in terms of our knowledge and the methods used to identify them.

### Fungi

#### Yeasts

In 1995, Espinet et al. identified a series of genes involved in cell growth and they demonstrated that 11 of them, from SHE1 to SHE11, do not have identifiable homologs outside of *Saccharomyces cerevisiae* (Espinet et al. 1995). These were the first examples of functional genes in yeast lacking homologs in other species. The term orphan was introduced by Bernard Dujon in *S. cerevisiae* in 1996, once the yeast genome project was accomplished (Dujon 1996). Comparative analysis between yeast sequences and the available genome sequences of other species at that time from various databases indicated that 25% of the *S. cerevisiae* genes that had no identifiable homologs and were referred to as orphan genes (Oliver et al. 1992). Later, in 2001, a study demonstrated that the SHE9 gene, which was initially considered an orphan gene, had a homolog in another yeast, *Candida albicans* (Andaluz et al. 2001). The study also showed that overexpression of this gene impairs cell growth in this species. Homology search was conducted with BLAST (McGinnis and Madden 2004) and the expression levels were estimated by Northern blot analysis. Two ATGATT hexamers were identified in the promoter region and, when present in the forward orientation, this hexamer exerts a positive regulatory control in response to cell proliferation. As this study showed a homolog for SHE9 gene outside of *C. albicans*, we could no longer consider this gene as an orphan gene for *S. cerevisiae*. Moreover, when we checked the Saccharomyces Genome Database (SGD), we noticed that only SHE1, SHE2 and SHE10 remain labeled as orphan genes. This shows how important depth is in a phylogenetic sampling to consider a gene orphan or not.

In 2008, an orphan yeast gene, BSC4, was identified and considered for the first time as a *de novo* emerged gene (Cai et al. 2008). Researchers performed a tBLASTN search, using the protein sequence of BSC4 against the genome sequences of 81 fungal species, including *Saccharomyces bayanus*, *S. kudriavzevii*, *S. mikatae*, *S. paradoxus* and *S. cerevisiae*, revealing that this gene is unique to *S. cerevisiae*. The absence of matches in the other species was confirmed by Southern Blot, where only *S. cerevisiae* showed a hybridization signal. Synteny analysis indicated that the flanking genes of BSC4 have their orthologs in the same syntenic blocks of *S. bayanus*, *S. mikatae* and *S. paradoxus*. This also revealed that the species other than *S. cerevisiae* contained multiple premature stop codons at the expected position of BSC4 gene. In light of this evidence, the authors concluded that BSC4 represents a case of *de novo* origin. Subsequent studies suggested that the expression of BSC4 expression increases when *S. cerevisiae* enters the stationary phase. Therefore, this gene may play a role in DNA repair and contribute to the evolutionary fitness of *S. cerevisiae* in nutrient-poor environments.

In 2010, another study built on the evidence for *de novo* gene emergence, identifying MDF1, a gene with a distinct regulatory function in yeast mating (Li et al. 2010b). The study showed that the protein-coding gene MDF1 arose *de novo* and can suppress mating efficiency. Firstly, the authors performed a BLAST search against the UniRef90 database and found no significant homologous ORF in the closely related species. They confirmed that the synteny was conserved in multiple fungal species, and then manually aligned the intergenic region between the flanking genes in other species. This region could not encode for proteins in any species other than *S. cerevisiae* due to the presence of stop codons and frame-shifting indels. The functional role of this *de novo* gene was assessed using an antisense transcript that acts as a transcriptional repressor, regulating MDF1 expression by binding to its promoter region. Microarray analysis showed that when the MDF1 gene expression was suppressed, mating success was significantly higher. By binding to a protein that is one of the determinants of yeast mating type, MDF1 suppresses yeast mating behavior and allows rapid vegetative growth.

In 2018, a more comprehensive research was conducted on 15 different yeast species, identifying 703 *de novo* gene candidates. The existence of 85 of these candidates was validated by proteomic data, and 25 showed evidence of translation based on mass spectrometry experiments (Vakirlis et al. 2018). The study suggested that *de novo* gene birth is a widespread phenomenon in yeast, although only a few of these genes are ultimately maintained by selection. To identify the 703 *de novo* genes, the authors first performed a multiple sequence alignment of the protein sequences for each family and constructed HMM and PSSM profiles. Then they performed exhaustive similarity searches against several databases, using BLASTP for singletons and PSI-BLAST for families with their respective HMM or PSSM profiles. They then took the families with no hits against nr, compared the families with no hits against nor between them, and merged the families with significant similarity. To distinguish between orphan genes that highly diverged from ancestral genes and *de novo* genes, the authors simulated the evolution of protein families using the ROSE program (Stoye et al. 1998) and inferred the branch of origin for each family along the genus phylogeny by phylostratigraphy using a custom pipeline. A simulated family that was assigned to the root of the focal genus was considered as a highly diverged gene, whereas one that was not was considered as a *de novo* gene. This was one of the first studies in yeast in which results were not simply obtained by BLAST or similar homology searches, but rather from a more comprehensive and detailed pipeline, including the use of HMMs.

In 2018, another study examined the spread and fixation of *de novo* genes in *S. cerevisiae* populations, revealing insights into their persistence. The research identified 84 *de novo* genes, some of which are expressed and translated under specific conditions (Wu and Knudson 2018). To identify these genes, the authors first performed a BLASTP search of the *S. cerevisiae* proteins against those of 20 other *Saccharomycetaceae* species. They excluded the genes for which they could not find the orthologous noncoding sequence in the outgroup genomes of *S. paradoxus* and *S. mikatae*. They confirmed the expression of the genes using transcriptomic data. They compared their results with three previous studies (Carvunis et al. 2012; Lu et al. 2017; Vakirlis et al. 2018) and found that only 33% of their *de novo* genes were shared with at least one of the other three studies. Surprisingly, there were no *de novo* genes common to all four studies. The authors explained these discrepancies by the exclusion of overlapping ancient genes for certain studies, *e*-value differences for homology searches, and different thresholds for required expression levels. They also suggest that one of the studies identified noncoding regions as homologs rather than only the protein-coding genes. Some genes were only expressed under specific conditions, which were not taken into account by one of the other studies. Indeed, 10% of the newly identified *de novo* genes were only expressed under specific conditions. This highlights the fact that different thresholds, scoring systems, and criteria, can lead to different sets of orphan and *de novo* genes identified.

Furthermore, the authors of this paper also compared the transcriptomic data between the wild type and two mutants, in which the products of the mutants were two proteins involved in pre-mRNA splicing and nonsense-mediated mRNA decay (Chapman and Boeke 1991; Gould et al. 2016). The results showed high expression levels for 8 *de novo* genes in the case of mutants which could be regulated by the mutant proteins and therefore they concluded that these *de novo* genes are possibly involved in mRNA processing. They also used ribosome profiling data to show that 51% of their *de novo* genes were found to be translated at specific time points or conditions. They then took advantage of several microarray data from SPELL database (Hibbs et al. 2007) which is a query-driven search engine for large gene expression microarray compendia. Results showed that among the 84 *de novo* genes, 87% were associated with 52 functional categories defined by SPELL. Overall, 73% of the genes were identified as involved in carbon utilization processes while 7% were involved in cell aging.

Another study, published in 2020, characterized a *de novo* gene YBR196C-A in *S. cerevisiae* (Vakirlis et al. 2020b), coding for a transmembrane protein. The orphan status of the gene was verified by the absence of homologs in other *Saccharomyces* and fungal species. Syntenic studies then revealed that this gene most likely emerged *de novo* from a thymine-rich intergenic region. Expression of this gene was shown to have a beneficial impact on yeast fitness. The authors verified the bioinformatics prediction of a transmembrane localization experimentally by using EGFP-tagged visualization of the protein via confocal microscopy and membrane association assays, which revealed the presence of the protein at endoplasmic reticulum (ER) membrane. Follow-up studies in 2023 and 2024 further characterized this *de novo* gene. Constructing a reference translatome for *S. cerevisiae* and using an experimental approach to mutate ATG to AAG codon in some strains, preventing ORF translation, Wacholder et al. revealed that YBR196C-A gene has phenotypic consequences when its translation is inhibited (Wacholder et al. 2023). There was a fitness reduction under stress conditions. The authors also highlighted the orphan status of other genes of *S. cerevisiae*, most importantly HUR1, ICS3, YPR096C and YDL204W-A. These genes are involved in DNA repair (Omidi et al. 2018), copper homeostasis (Alesso et al. 2015), regulation of a gene involved in sugar metabolism (Hajikarimlou et al. 2020) and cell fitness, respectively (Houghton et al. 2024). Confirming initial analyses, Saeki et al. explicitly identified the YBR196C-A gene as encoding a beneficial emerging protein (BEP) localized at ER using overexpression profiling experiments (Saeki et al. 2023 ). Houghton et al. re-analyzed fitness measurements from the 2020 study and showed that ER-localized BEPs all contain transmembrane domains followed by short C-termini (Houghton et al. 2024). They also showed the pathways that this protein might be involved in and revealed that ER-localized BEPs are beneficial across more conditions than other BEPs. Given all these evolutionary and experimental studies, we can assume that YBR196C-A is one of the best-characterized *de novo* genes so far, even though the function of this gene is not yet fully understood.

A review paper specifically focused on *de novo* genes in yeast published in 2022 also describes some of the above-mentioned cases and the methodology used to detect and study the function of such genes in yeasts (Parikh et al. 2022).

#### Other Fungi

In 2015, Kohler et al. conducted a comparative genomics analysis to elucidate the evolution of the mycorrhizal lifestyle in fungi and determined that 7% to 38% of the genes induced during symbiosis are orphan genes, many of which encode secreted effector-like proteins (Kohler et al. 2015). The study involved sequencing the genomes of 13 ectomycorrhizal (ECM), orchid (ORM), and ericoid (ERM) fungal species, along with 5 saprotrophic species, and comparing them with existing fungal genomes with Markov Cluster Algorithm (MCL). The gene expression of identified genes was assessed with RNA-seq. These findings suggest that the evolution of mycorrhizal symbiosis in fungi occurred through convergent evolution, leading to the emergence of distinct sets of genes that are specifically activated during mycorrhizal interactions in different fungal lineages. In contrast to most of the previously described methods to identify orphans in yeast, in this more recent study, MCL algorithm was used for the comparative genome analysis.

In 2016, another study investigated the evolution of orphan genes in the genome of *Zymoseptoria tritici*, a fungal pathogen of wheat. The authors identified 296 such genes in the *Z. tritici* genome (Plissonneau et al. 2016). Utilizing long-read genome sequencing, genetic mapping, and transcriptomics, they assembled and annotated the genome of the virulent *Z. tritici* field isolate 3D7. Comparative analyses with the reference genome IPO323 of the same species using BLASTn and synteny analysis revealed significant chromosomal inversions and variations in transposable element clusters, leading to extensive chromosomal-length polymorphisms. Notably, both genomes contained large, unique sequence tracts with the 3D7 genome harboring 296 genes absent in IPO323. These orphan genes were enriched in putative effector genes, including one highly upregulated during wheat infection. However, the paper does not state that these 296 genes considered orphans are missing in other fungal species or other species in general. They only compared their genome to the reference genome, which is IPO323. Therefore we cannot conclude for sure that *Z. tritici* has 296 orphan genes as there might be gene loss cases in IPO323 as well.

Continuing the exploration of orphan genes in fungal pathogens, a 2020 study on *Fusarium graminearum* identified an orphan protein that actively modulates host immunity (Jiang et al. 2020). The authors used BLASTp for protein homology search and also tBLASTn to search against genomes, firstly to two closely related *Fusarium* species and if they were orphan, they were compared also against nr. They identified a total of 971 (∼7.3% of all protein-coding genes) orphan genes. The authors then focused on one of these orphan genes which were predicted to encode a protein with a signal peptide for secretion, Osp24. According to protein interaction assays, this protein, which is unique to *F. graminearum*, appears to facilitate infection by targeting TaSnRK1α, a key regulator of the plant's immune response. The researchers demonstrated that the orphan protein interacts with TaSnRK1α by targeting it for degradation through the proteasome pathway, thereby weakening the plant's immune defenses.

Also in 2020, other researchers investigated the emergence of new gene families in another fungal genus, *Amanita*, focusing on their association with the evolution of ECM symbiosis and the study identified 109 gene families unique to ECM *Amanita* species, absent in closely related asymbiotic species (Wang et al. 2020). These unique gene families were found to be under strong purifying selection and upregulated during symbiosis, suggesting their functional relevance to the mutualistic association. Among the unique gene families, the most upregulated gene in symbiotic cultures encodes a 1-aminocyclopropane-1-carboxylate deaminase, an enzyme capable of downregulating the synthesis of the plant hormone ethylene, a common negative regulator of plant-microbial mutualisms. Furthermore, the homology search and synteny showed 2 of these orphan gene families are candidate *de novo* gene families, with so far no known function.

In late 2022 and 2023, Wang et al. studied *Neurospora crassa* lineage-specific genes, revealing 670 orphan genes (Wang et al. 2022, Wang et al. 2023a). They then showed that gene duplication, relocation, and regional rearrangement drive this process (Wang et al. 2023b). They used a phylostratigraphic approach and BLAST search against FungiDB to identify these gene clusters, and then verified their expression via transcriptomic data. By analyzing synteny and clustering patterns, they found that 78% of these clusters are near telomeric regions with extensive noncoding DNA and duplicated genes. These regions, termed “rummage regions,” constitute a favorable environment for new genes to arise and evolve. Using transcriptomics from 68 data points, the researchers found that these genes often have peripheral regulatory functions, though they play critical roles under specific conditions. The study highlighted mas-1, a lineage-specific orphan gene likely from a lysophospholipase precursor, which contributes to cell wall integrity and antifungal resistance.

Aside from their roles in adaptation and symbiosis, orphan genes have also proven useful as molecular markers for species identification. A 2022 study developed an approach to distinguish *Aspergillus* species using orphan genes (Wang et al. 2022, Wang et al. 2023a). The researchers developed a multiplex PCR method to identify *Aspergillus cristatus* and *Aspergillus chevalieri* in Liupao tea using species-specific orphan genes In this study, six fungal strains were isolated from Liupao tea and identified as *A. cristatus*, *A. chevalieri*, and *A. pseudoglaucus*. According to this study, traditional ITS sequencing proved insufficient to distinguish closely related species due to high sequence conservation. To overcome this, the researchers used comparative genomics to identify orphan genes unique to each species and designed species-specific primers for multiplex PCR. This approach enabled rapid and accurate identification of *A. cristatus* and *A. chevalieri* in both Liupao and Fu brick teas, highlighting the utility of orphan genes in distinguishing closely related species.

### Animals

#### Drosophila and Other Insects

In 2000, a study of the model fly species *Drosophila melanogaster*, nematode species *Caenorhabditis elegans* as well as humans showed that about 30% of *D. melanogaster* genes had no identifiable homologs and were therefore considered orphans according to BLASTP results (Rubin et al. 2000). Then, in 2003, another study followed up to investigate whether there was a change in the proportion of predicted orphan genes over time in *Drosophila* and compared about 14,000 predicted proteins of the *Drosophila* proteome with other insects using BLASTP (Domazet-Loso and Tautz 2003). The authors compared the different results obtained with different e-values varying from 10^−100^ to 10 and, as expected, the number of sequences with no homologs is very small at the highest *e*-values due to many insignificant random matches. The results for more stringent lower *e*-values, the ones preferred by many studies, 10^−3^ to 10^−5^, showed that there were still 26% to 29% of *D. melanogaster* genes that had no identifiable homologs. The results thus indicated that there was no significant change in the proportion of orphans, despite the growth of the database and improvements in annotation over time. To be sure that this *e*-value range was the best choice, they compared the different homologs obtained at different *e*-values and concluded that 10⁻³ to 10⁻⁵ range as the optimal balance between false positives and true orphans. This *e*-value range is still the most used in most of the studies. The authors then carried out a comparative analysis of expressed genes only between *D. melanogaster* and *D. yakuba* and the results showed 8.4% and 19.7% of orphan genes for *D. melanogaster* were expressed for the embryonic and adult stages respectively. Compared with the whole-genome analysis, these values were significantly lower. The study suggested that this could be due to incorrect annotation at the genomic level, or that orphans are likely to be expressed at lower levels than nonorphan genes. Incorrect annotations can be problematic because they may lead to the misidentification of genes, causing some genuine orphan genes to be overlooked or misclassified (Weisman et al. 2020). This can result in an underestimation of their prevalence and functional significance. Also, it is important to note that some genes might be expressed only at certain life stages. Finally, the researchers concluded that *D. melanogaster* contains an important number of orphan genes even in the light of new data and the selection of e-value is important, with the recommended range being between 10^−3^ and 10^−5^.

Early studies focused on the proportion of orphan genes in the genome, but subsequent research attempted to assess their biological significance as well, particularly in reproduction. In 2006, a study described five *de novo* genes expressed in the testes and implicated in male production in *D. melanogaster* under selective pressure (Levine et al. 2006). First, the authors identified orphan genes by BLASTN against the genomes of two other *D. melanogaster* species and retained only those that had complete cDNA sequences according to the Flybase database and/or those that were experimentally confirmed. They then applied syntenic approaches and retained only five genes with high-quality syntenic alignments of the flanking regions of the *de novo* gene in *D. melanogaster* compared with *D. yakuba, D. erecta*, and *D. ananassae*. Southern blot analysis confirmed their computational prediction. The authors concluded that there were five *de novo* genes in *D. melanogaster* that met their stringent criteria, suggesting that there were probably many more. RT-PCR data from RNA isolated from whole adult male and female reproductive tissues showed that all five genes were expressed in the testes. Four of the five *de novo* genes demonstrated X-linked expression. In 2007, a follow-up study showed that *D. yakuba* and/or *D. erecta* also have 7 additional *de novo* genes involved in male reproduction (Begun et al. 2007). They analysed the *D. yakuba* testis-derived cDNA library and followed a similar procedure to the previous study that identified *D. melanogaster de novo* genes. They concluded that *de novo* gene birth is an important phenomenon for male reproduction in *Drosophila* species. A subsequent study conducted in 2014 provided further evidence that a greater number of testis-expressed *de novo* genes are involved in male reproduction in *D. melanogaster* by examining different populations of this species (Zhao et al. 2014). An Illumina paired-end RNA sequencing approach was employed to characterize the testis transcriptome of six previously sequenced *D. melanogaster* strains. The resulting analysis revealed that there are a total of 142 expressed *de novo* genes in the testis even under the very strict filtering criteria. In 2021, a study also demonstrated experimental evidence of implication of a *de novo* gene in *D. melanogaster* where the gene is required for spermatid chromatin condensation (Rivard et al. 2021).

While most *de novo* gene studies in *Drosophila* have identified links with male reproductive functions, one study identified a *de novo* gene involved in female reproduction, expanding the known functional repertoire of orphan genes in this species. Similar approaches to those employed in other recent studies were used, including BLAST for homology search, synteny to detect noncoding regions of the *de novo* gene in closely related species, and expression levels in different tissues for the identified gene (Lombardo et al. 2023).

Whereas previous studies examined species-specific *de novo* genes in *Drosophila*, later research expanded the scope to investigate orphan genes across multiple species within the genus, providing insights into broader evolutionary trends. In 2020, another group of researchers who had been investigating orphan genes and *de novo* gene birth in *Drosophila* demonstrated that across 12 *Drosophila* species, there are 6,297 orphan genes, with between 8.7% and 39.2% of them resulting from *de novo* gene birth (Heames et al. 2020). To identify them, the authors first clustered all sequences of the 12 *Drosophila* species and 3 outgroup species by BLASTP and then they compared the clusters to the NCBI nonredundant (nr) database. Furthermore, a phylostratigraphic method was employed to ascertain the gene gain timing scenarios, while syntenic approaches were utilized to detect instances of *de novo* gene birth within the *Drosophila* clade. Here, it is important to underline that the study was not describing species-specific orphan genes like the previous ones but it was revealing orphan genes at the whole *Drosophila* genus level.

Beyond identifying orphan genes, researchers have also sought to understand their structural properties and evolutionary stability. One such study focused on the structural characterization of the Goddard protein, a *de novo* gene involved in *Drosophila* male fertility (Lange et al. 2021). To achieve this, the researchers employed a combination of modeling, NMR and circular dichroism approaches, which revealed that the protein in question contains a central α-helix, while the remaining portions are predominantly disordered. The researchers demonstrated that this structure is a novel one by comparing the obtained structure to the PDB database. Furthermore, they proposed that this structure has been preserved by the organism over millions of years, as evidenced by its conservation across diverse *Drosophila* species (but absence from the rest of species). To substantiate this hypothesis, they reconstructed the ancestral sequence of the node shared by five *Drosophila* species that express this protein and utilized the structure that they described for each of them to infer an ancestral structure. Additionally, they demonstrated that this protein localizes to elongating sperm axonemes and that its absence impairs the individualization of elongated spermatids.

Expanding on individual cases like Goddard, recent large-scale analyses have examined the structural evolution of *de novo* proteins in *Drosophila*, offering insights into their folding and functional constraints. In 2024, a study identified 555 *de novo* proteins in *D. melanogaster* by using homology and synteny approaches similar to other studies (Peng and Zhao 2024). Furthermore, they employed AlphaFold2, ESMFold and RoseTTAFold to predict structures, and demonstrated that the majority of these structures are either partially folded or unstructured, as indicated by low pLDDT scores for confidence from each of the three tools. However, they also described several well-folded structures. It is noteworthy that the ancestral sequence reconstruction suggested that these well-folded *de novo* proteins were already well-folded at the time of their origin. Furthermore, a comparison with the PDB database revealed that most of these well-folded *de novo* proteins adopt existing folds, despite the low sequence identity between the sequences responsible for their construction. However, it must be highlighted that these structure prediction methods depend on multiple sequence alignments or they are trained with homologous proteins. Therefore, limitations are expected for the prediction of orphan protein structures which, by definition, lack homologs.

Overall, in *Drosophila*, numerous studies have explored orphan and *de novo* genes, and their functional characterization has been mainly associated with reproduction or sex determination. While many orphan genes have been identified, functional validation remains a challenge, emphasizing the need for further studies beyond reproductive traits.

In 2013, Wissler et al. conducted a large-scale comparative genomic analysis to investigate the mechanisms and dynamics of orphan gene emergence in insect genomes, with a particular focus on ants (*Formicidae*) (Wissler et al. 2013). The study revealed that orphan genes make up a substantial fraction of insect genomes, ranging from 10% to over 30% depending on the species analyzed. A key finding was that *de novo* gene birth appears to be the predominant mechanism in *Formicidae*: *de novo* origin accounted for 43.5% to 61.2% of species-specific orphan genes, far exceeding divergence after gene duplication (6.4% to 9.9%) and other mechanisms. The distribution of orphan genes appeared to be largely random across the genome, suggesting widespread and independent emergence events. Notably, several orphan genes exhibited specific expression profiles across tissues or developmental stages, supporting their potential role in lineage-specific traits and ecological adaptations.

In 2021, a group of researchers investigated orphan genes in the diamondback moth *Plutella xylostella*. They found two functional orphan genes via RNA interference (RNAi) and gene expression analyses (Li et al. 2021). RNAi silencing of these genes reduced sperm count and motility, significantly impairing male fertility. Further analysis showed these genes are highly expressed in the testes, with one gene showing expression patterns consistent with late-stage spermatogenesis. These findings suggest these genes contribute to male reproductive success and are under strong selection pressures due to their roles in sperm function. This highlights the importance of orphan genes in species-specific reproductive adaptations in *P. xylostella*. Another study in 2024 described another orphan gene in the same species that enhances male reproductive success (Zhao et al. 2024b). The authors demonstrated that this orphan gene, lushu, encodes a sperm protein. Through CRISPR/Cas9-generated mutants lacking this gene, they found males exhibited reduced fertility, with lower sperm viability and motility. Expression analysis showed lushu is highly active in the testes, suggesting a role during sperm maturation. This gene's location on the Z chromosome and its high prevalence in different *P. xylostella* populations suggest it may be under strong selective pressure, likely evolving to meet reproductive demands specific to this species, similar to *Drosophila*.

#### Nematoda

In 2015, Mayer et al. investigated the role of an orphan gene named dauerless in the *Pristionchus pacificus* necromenic and predatory nematodes, specifically its regulation of dauer development and intraspecific competition (Mayer et al. 2015). The dauer stage is a stress-resistant, nonfeeding larval stage in nematodes that allows survival under harsh environmental conditions such as overcrowding or starvation where the metabolism and development are in pause. The study revealed that the *dauerless* gene influences the dauer formation process. The researchers showed that CNV in the dauerless gene plays a crucial role in regulating the nematode's ability to enter or bypass the dauer stage by several experiments and RNA-seq data. Nematodes with higher copy numbers of the dauerless gene were more likely to suppress dauer formation, which in turn gave them a competitive advantage in environments where resources were limited. This study highlights how CNV in an orphan gene can drive intraspecific competition and influence survival strategies in nematodes.

Following this finding, a study in 2016 described the retroviral origins of an orphan gene, F58H7.5, in *Caenorhabditis elegans* (Kapulkin 2016). While the gene's orphan status was confirmed through direct homology searches, which demonstrated the absence of detectable homologs in other species, the author conducted a comprehensive investigation into its retroviral origins. The study traced the gene back to a potential retroviral insertion, thereby suggesting that exogenous viral elements may have contributed to its emergence within the nematode lineage. Supporting evidence was provided for this hypothesis by identifying sequence similarities between the orphan gene and known retroviral elements, focusing on structural motifs that are typically associated with viral proteins. Furthermore, the integration site of the gene was investigated, demonstrating that the surrounding genomic region exhibited hallmarks of retroviral insertions, including long terminal repeats (LTRs) and flanking sequences commonly associated with viral integration events. These findings provide compelling evidence for the gene's retroviral origin, elucidating the manner in which viral genetic material was likely co-opted and repurposed for functional use in *C. elegans*. Overall, this constitutes a case of lineage-specific horizontal acquisition of a retroviral element eventually leading to the emergence of an orphan gene lacking homology in other nematodes.

In 2019, another study on *C.elegans* identified 893 orphan genes specific to this species, demonstrating that 4.4% of its protein-coding genes lack homologs in other species (Zhang et al. 2019). Among these, the researchers determined that six genes originated *de novo*. To identify orphan genes, a BLASTP search against closely-related species was performed, which was followed by a BLAST search of coding sequences (CDS) to locate possible noncoding regions in closely related species to be able to identify *de novo* gene candidates. In the identified noncoding regions, the authors searched for the presence of alternative start and stop codons and verified synteny to confirm these candidates as *de novo* genes. Then, similar to previous studies, they verified the expression of these genes via transcriptomic and translation via proteomic data. This multi-step approach allowed them to characterize these genes as recent additions unique to the *C. elegans* lineage, highlighting the potential for *de novo* gene birth in driving species-specific adaptations. The authors found that the expression levels of *de novo* genes are predominantly very low in restricted developmental stages and tissues, but 50% of the identified *de novo* genes showed detectable expression in the dauer stage. Moreover, the study revealed that an important part of these genes were expressed in gonads in adult tissues, which suggest a role in reproduction.

In the same year, Lightfoot et al. uncovered a self-recognition mechanism in *P. pacificus* that prevents cannibalism among its offspring (Lightfoot et al. 2019). The study identified an orphan gene encoding a small peptide, SELF-1, which allows *P. pacificus* to recognize its progeny and avoid consuming them. Through behavioral assays, the researchers demonstrated that *P. pacificus* selectively avoided predation on its own larvae while attacking unrelated larvae, implicating SELF-1 in self-recognition. SELF-1, a 63-amino acid peptide located on the larval surface, has a hypervariable C-terminal region crucial for its function; even a single amino acid change in this region disrupts recognition, leading to cannibalistic behavior. When examining homologs in other nematodes, the team identified SELF-1 as a taxon-restricted orphan gene, suggesting that it either evolved rapidly within *P. pacificus* or emerged *de novo*, providing a unique evolutionary adaptation to enhance survival strategies in competitive environments. This study represents one of the first explorations of orphan genes in behavioral adaptations, with SELF-1 as an example of a gene driving intraspecific recognition.

Later in 2019, another study investigated the whole set of orphan genes in the *Pristionchus* genus (Prabh and Rödelsperger 2019). Using comparative genomics and phylostratigraphy, the authors revealed that in each *Pristionchus* species, approximately 10% of all genes lack homologs in any other species and can be considered orphans. At the genus level, 70% of the diplogastrid-specific orphan genes are shared by at least two different *Pristionchus* species. Among these, they identified 29 high-confidence species-specific orphan genes in *P. pacificus*, two of which were shown to have emerged *de novo*. To identify these *de novo* genes, the researchers employed tools such as CYNENATOR (Rödelsperger and Dieterich 2010) for synteny analysis and Exonerate (Slater and Birney 2005) for mapping orphan proteins to the genomes of closely related species. Even though they did not provide functional insights, the authors hypothesized that these species-specific genes may contribute to this nematode's ability to thrive in specific environmental niches. Again, it is important to note that the study identified species-specific orphan genes as well as genus-specific ones therefore this must be taken into account when comparing to other studies.

In 2021, Rödelsperger et al. expanded on their research on *P. pacificus*, demonstrating that sperm cells are a source of genomic novelty and rapid evolution in this species, similar to patterns observed in *Drosophila* (Rödelsperger et al. 2021). This study utilized spatially-resolved transcriptome data to map gene expression across distinct anatomical regions in adult nematodes, revealing that sperm cells exhibited particularly high levels of novel gene activity and rapid gene evolution. The authors suggested that many of these novel genes correspond to highly diverged or *de novo* orphan genes identified in their previous research, proposing that sperm-specific regions could drive evolutionary innovation in nematodes by fostering the emergence of new, adaptive genes. Moving on in 2022, Prabh et Rödelsperger also analyzed gene turnover rates in *P. pacificus* to explore the evolutionary dynamics of *de novo* genes compared with duplicated genes (Prabh and Rödelsperger 2022). By sequencing six diverse strains, the study investigated how different origins of genes—*de novo* formation versus duplication—affect their evolutionary persistence and rates of loss. The researchers found that *de novo* genes, aligning with a rapid turnover hypothesis, experience faster rates of both gain and loss. The study highlighted that *de novo* genes remain under weak evolutionary constraints and tend to disappear or evolve rapidly, especially in young age classes. In contrast, duplicated genes showed greater stability and longer retention across evolutionary time scales. These findings suggest that *de novo* genes contribute to genomic innovation, albeit with high rates of attrition, emphasizing the role of gene turnover in shaping *P. pacificus* adaptability and diversity over time.

In 2022, a new study on *C. elegans* uncovered intraspecific *de novo* gene birth by analyzing presence–absence variants (PAVs), a novel approach for identifying genes that are specific to certain strains but absent in others (Lee et al. 2022). This study represents a shift from traditional interspecies comparisons to intraspecies analyses, allowing the researchers to capture recently emerged genes within the *C. elegans* lineage. Using long-read sequencing technology, the authors studied the genomes and transcriptomes of two strains, *CB4856* and *PD1074*, and identified 46 species-specific genes unique to these strains, many of which are likely *de novo* genes. By employing BLAST and LiftOver (Genovese et al. 2024) for precise gene localization, they confirmed that these genes were either newly formed or lost in the other strains.

#### Humans and Other Vertebrates

The pioneering studies in model species such as yeast and *Drosophila* demonstrated that their genomes comprise a substantial number of orphan genes, which perform a variety of functions. This further motivated researchers to study orphan genes in humans and other vertebrates as well. In 2010, a study demonstrated that FLJ33706, an orphan gene according to the evolutionary biology definition of this review, which emerged *de novo*, is associated with human brain functions (Li et al. 2010a). The expression of this gene in the brain was confirmed by RT-PCR analysis in multiple tissues, and its orphan status was verified through homology searches against the nr and uniref databases. Subsequently, syntenic genome alignments confirmed that this is a human-specific orphan gene that emerged *de novo*. Furthermore, the study demonstrated that this gene is overexpressed in the brains of individuals with Alzheimer's disease (AD), once again through RT-PCR analysis on 18 healthy brains and 20 AD brains. This identified gene constituted the inaugural example of a *de novo* gene in humans, exhibiting substantial evidence for a function in the brain.

While the 2010 study identified a single *de novo* gene associated with human brain functions, researchers soon expanded their scope to identify *de novo* genes on a genome-wide scale. In 2011, a group of researchers sought to determine the total number of *de novo* genes in humans. They identified 60 such genes (Wu et al. 2011). To identify them, they searched all human proteins against the sequences of other primates and identified 584 human-specific orphan genes. They excluded the ones that did not have start or stop codons in humans and then they performed BLAST analysis against chimpanzee and orangutan genomes with the remaining 352 orphan genes. Then, they identified the ones that had potentially translatable open reading frames and if these regions were disrupted in chimpanzee or orangutan (presence of stop codons, frame-shift indels, bad start codons) via a custom pipeline. Finally, they described 60 *de novo* genes, including FLJ33706 from the 2010 study of the brain. Moreover, the expression levels of these genes in humans, as determined by RNA-seq data on diverse tissues, indicated that the majority of these genes exhibit elevated expression in the cerebral cortex and testes. This observation suggests that these genes may contribute to traits that are exclusive to the human species.

Beyond their potential roles in brain development or reproduction, some orphan genes have been shown to be implicated in disease processes. One notable example is PBOV1, a *de novo* gene linked to cancer progression. In 2013, a study revealed the presence of this gene, with tumor-specific expression particularly in prostate and breast cancers (Samusik et al. 2013). To identify PBOV1 as a *de novo* gene, the authors performed a comparative genomic analysis using MULTIZ multiple genome alignments available from the UCSC Genome Browser to compare the PBOV1 protein-coding sequence (CDS) across 34 genomes of placental mammals. This comparative alignment allowed them to map homologous regions and identify mutations in humans that resolved frame-shift and stop codons disrupting the ORF in nonhuman species. They then assessed the alignment between human PBOV1 and other mammalian genomes by calculating the fraction of the human CDS that could be aligned to each species. In placental mammal species such as Laurasiatheria and Glires, the ATG start codon and a 12-base-pair region is missing, producing a frame-shift deletion, yielding sequences incapable of producing a similar protein. The genomic analysis showed that while over 99% of the human PBOV1 sequence could be aligned with primate genomes, in nonhominid primates, an early stop codon restricted the protein similarity to 80% of its length. However, this stop codon was mutated in the common ancestor of hominids, restoring the ORF and allowing the gene to evolve into a functional protein in humans. Then, similar to other studies, RT-PCR analysis on different tissues revealed that this *de novo* gene is expressed in important part of the cancer types; including breast cancer, cervical, ovary and endometrial cancer, lung cancer, nonHodgkin lymphomas, meningioma and seminoma. Using publicly available microarray datasets, the researchers also found that high levels of PBOV1 expression in breast cancer and glioma samples were significantly associated with positive clinical outcomes. Interestingly, PBOV1 expression was observed in primary but not recurrent high-grade gliomas, suggesting a negative selection against PBOV1-expressing cancer cells.

In 2015, another study revealed 634 human *de novo* genes using BLAST for homology search and synteny for the verification of the *de novo* status (Ruiz-Orera et al. 2015). The analysis of the patterns of tissue expression in assembled transcripts demonstrated that the majority of these genes were expressed in the testis. Conversely, only a few were expressed in the brain, liver, and heart. Consequently, the researchers concluded that *de novo* genes were twice as likely to exhibit testis-restricted expression compared with the rest of the genes in humans.

Then, in 2016, Guerzoni et al. (Guerzoni and McLysaght 2016) investigated the *de novo* emergence of genes in the primate lineage, revealing a slow but consistent rate of new gene formation over evolutionary time. The study utilized similar methods to previous ones to identify *de novo* gene candidates across multiple primate genomes, particularly great apes such as humans, chimpanzees, orangutans and gorillas. By examining coding and noncoding regions for sequence homology and structural alignments, the authors identified genes with no clear ancestral counterparts in closely related species, establishing their *de novo* origin. One of the key findings was that some *de novo* genes had experienced incomplete lineage sorting (ILS). For instance, in some cases the *de novo* gene was present in humans and gorillas, while in chimpanzees, this is the ancestral noncoding regions that were retained at the same locus. This ILS phenomenon was notably present in genes that showed tissue-specific expression in humans, particularly the brain, suggesting an adaptive role in traits unique to primates. Such instances of ILS suggest *de novo* genes may initially have a neutral effect on fitness and experience a long period of polymorphism prior to fixation. This paper was another example of high impacts of methodology used to identify *de novo* genes. Indeed, the researchers compared their results with those of Ruiz-Orera et al. (2015) but found no overlap in the *de novo* gene candidate lists. This is largely explained by filtering-out of intronless genes in the former study, while such genes constitute nearly half of the cases in the new study. The other half is mainly explained as regions not annotated as genes in the version of the databases used in the more recent study.

Moving on, in 2022, Vakirlis et al. (Vakirlis et al. 2022) described the *de novo* birth of functional microproteins in humans. The study focused on microproteins originating from small open reading frames (sORFs), which are known to have significant fitness effects. To trace their evolutionary origins, the authors performed a comparative analysis across 99 vertebrate species. They reconstructed phylogenetic trees and ancestral sequences to determine when each sORF emerged. If an ancestor lacking an intact ORF was found to precede those with an intact ORF, the ORF was classified as having originated *de novo*. Expression of the *de novo* sORFs was then confirmed using transcriptomic data. Ultimately, the study identified 155 *de novo* microproteins, of which 44 had significant fitness effects, indicating a role in human biological functions. Notably, two of these microproteins likely emerged after the human-chimpanzee split, suggesting roles in human-specific traits and evolution.

In 2023, a group of researchers identified 74 *de novo* genes with long noncoding RNA (lncRNA) origins that play unique roles in human brain development (An et al. 2023). The study concentrated on the evolutionary transition of lncRNAs into protein-coding genes through mechanisms such as RNA splicing and nuclear export. By employing comparative genomics and experimental verification (mass spectrometry and RNA-seq) in human cortical organoids and transgenic mice, the researchers demonstrated that 45 of these genes are human-specific, whereas the remainder are hominoid-specific, having evolved subsequent to the divergence from rhesus macaques. The *de novo* genes were found to contribute to key human brain traits, including cortical development and brain size expansion, thereby emphasizing their potential role in shaping human-specific cognitive abilities. Later on in 2024, a study from Leushkin and Kaessmann contradicted and critically re-evaluated the findings (Leushkin and Kaessmann 2024). The re-analysis, utilizing various genomic resources and extensive ribosome profiling data, revealed that SMIM45 is, in fact, a mis-annotated part of an ancient and longer vertebrate gene starting just upstream. The authors also identified issues with some of the remaining loci, indicating that most do not correspond to hominoid-specific *de novo* genes. This study underlined again the necessity for rigorous validation in orphan and *de novo* gene research to accurately determine the origins and evolutionary significance of these genes.

In 2024, another study conducted a comprehensive analysis to identify and characterize human orphan genes across multiple tissues and diseases (Singh et al. 2024). Using extensive RNA-seq data, a custom pipeline and phylostratigraphy, the researchers discovered thousands of highly expressed transcripts that did not correspond to any previously annotated genes. Approximately 80% of these transcripts contained ORFs with the potential to encode proteins unique to humans. The authors validated these findings using independent strand-specific and single-cell RNA-Seq datasets which confirmed the expression of these novel transcripts. Further differential expression analysis revealed that many of these orphan genes are dynamically regulated, exhibiting selective accumulation in specific tissues, cell types, developmental stages, tumors, and in response to conditions such as COVID-19. In addition, survival analysis indicated that hundreds of these novel transcripts overlapped with deleterious genomic variants, and thousands showed significant associations with disease-specific patient survival, suggesting their potential as diagnostic biomarkers or therapeutic targets.

Lastly, in a recent study in 2024, an investigation was conducted into the evolution of ORFs derived from a single gene, which are separated by a transcriptional silencer. The study demonstrated that one of these ORFs has emerged *de novo* and is likely to play a role in human brain development, as it is one of the identified *de novo* genes in the previous study (Delihas 2024). The non-*de novo* ORF has ancient origins, dating back approximately 462 million years, and is present across different species. The absence of homology has been verified, and the synteny with mouse has shown that at the same position, mouse only has the non-*de novo* ORF. The study also suggested that the transcriptional silencer in between them likely regulates the *de novo* ORF, which provides important evidence of a possible function.

Besides humans and other primates, orphan genes have also been studied in other mammals such as mice and other vertebrates such as teleost fish.

In 2022, Petrzilek et al. examined the *de novo* emergence, existence, and eventual loss of the gene D6Ertd527e in murine rodents, shedding light on the high turnover rate of *de novo* genes within this lineage (Petrzilek et al. 2022). The researchers used CRISPR-Cas9 gene editing to delete the D6Ertd527e gene in *Mus musculus* to assess its functional role, specifically targeting the gene's coding regions to produce knock-out models. This deletion resulted in fertile mice with smaller litter. They also conducted RNA-seq across multiple murine species to analyze gene expression, focusing on D6Ertd527e's presence in oocytes and other reproductive tissues. These transcriptomic analyses revealed species-specific expression patterns, suggesting variability in the gene's adaptive significance. Visualization of RNA-seq data helped to map and confirm expression differences between *M. musculus* and other rodents. This approach illustrated how *de novo* genes, although potentially adaptive, can be short-lived under shifting evolutionary pressures, demonstrating D6Ertd527e's emergence and gradual loss within specific rodent lineages.

In 2014, antifreeze glycoprotein genes (AFGPs) in codfishes were studied by Zhuang and it was revealed that codfish AFGPs are orphans and likely have originated from noncoding DNA according to synteny (Zhuang 2014). Then in 2018, another study examined this origin and evolutionary pathway of AFGPs, particularly in the Atlantic rod codfish *Gadus morhua* (Baalsrud et al. 2018). The authors found that AFGPs likely emerged around 13 to 18 million years ago from noncoding DNA—a remarkable example of *de novo* gene birth. This development coincided with the onset of freezing temperatures in the Northern Hemisphere, supporting the hypothesis that AFGPs provided a survival advantage under extreme conditions. The study employed whole-genome sequencing and comparative genomic analysis using BLAST to trace the origins and distribution of AFGP genes, identifying these genes’ presence in multiple codfish lineages and variations in copy numbers across species. They noted a concentration of antifreeze functionality in the sequences, likely evolving from short repetitive tripeptide sequences found in noncoding regions that were repurposed into functional protein sequences for ice-binding. Furthermore, in species exposed to more severe freezing, codfishes show higher copy numbers of AFGP genes, indicating copy number variation as an adaptation to environmental demands. Later on in 2019, another study focused this time on another codfish family, Arctic cod (*Gadidae*) (Zhuang et al. 2019). The researchers found that a short sequence of noncoding DNA underwent repeated duplications, forming a tripeptide repeat sequence (threonine-alanine-alanine) that could bind ice crystals in the blood. Additional events followed: a single nucleotide deletion allowed for proper protein processing and secretion, and a translocation or insertion event provided the transcriptional signals necessary for gene expression regulation.

## Discussion and Conclusion

The study of orphan and *de novo* genes is a critical area of evolutionary and functional genomics, providing insights into lineage- and species-specific adaptations and biological innovation. These genes have been found with different prevalence in various animal and fungal species. Most research to date has been conducted on model organisms like *Drosophila*, *S. cerevisiae*, *C. elegans*, and humans, providing a clearer understanding of their prevalence and functional roles (Fig. 2). Studies on less explored organisms have also provided valuable insights into the evolution and potential functions of these genes. Therefore, while orphan genes are better understood in model species, this does not imply that they are absent or without importance in other species—rather, they have simply received less attention. A summary of orphan genes with known or possible functions can be found in Table 1.

**Table 1:** Examples of orphan and *de novo* genes with possible known functions

Gene or Gene Set	Species/Genus	Orphan status	Possible Function	Reference
SHE genes	*S. cerevisiae*	Orphan	Cell growth (partial)	Espinet et al. (1995)
BSC4	*S. cerevisiae*	*de novo*	DNA repair in stationary phase	Cai et al. (2008)
MDF1	*S. cerevisiae*	*de novo*	Suppression of mating	Li et al. (2010b)
YBR196C-A	*S. cerevisiae*	*de novo*	transmembrane protein in ER, involved in fitness.	Vakirlis et al. (2020b), Wacholder et al. (2023), Saeki et al. (2023), Houghton et al. (2024)
HUR1	*S. cerevisiae*	Orphan	Involved in DNA repair	Omidi et al. (2018), Wacholder et al. (2023)
ICS3	*S. cerevisiae*	Orphan	Involved in copper homeostasis	Alesso et al. (2015), Wacholder et al. (2023)
YPR096C	*S. cerevisiae*	Orphan	cell fitness (regulates a gene involved in sugar metabolism)	Hajikarimlou et al. (2020), Wacholder et al. (2023)
YDL204W-A	*S. cerevisiae*	Orphan	Cell fitness	Wacholder et al. (2023), Houghton et al. (2024)
Symbiosis-induced genes	ECM fungi	Likely mixed	Symbiosis establishment	Kohler et al. (2015)
296 unique genes	*Z. tritici*	Orphan	Infection-related	Plissonneau et al. (2016)
Osp24	*F. graminearum*	Orphan	Suppression of wheat immunity	Jiang et al. (2020)
Lineage-specific genes	*N. crassa*	Lineage-specific orphans, some likely *de novo*	Reproduction, cell wall integrity	Wang et al. (2022, 2023a, 2023b)
5 *de novo* testis genes	*D. melanogaster*	*de novo*	Male fertility	Levine et al. (2006)
7 *de novo* testis genes	*D. yakuba/erecta*	*de novo*	Male fertility	Begun et al. (2007)
142 *de novo* testis genes	*D. melanogaster*	*de novo*	Male fertility	Zhao et al. (2014)
Female reproductive tract *de novo* gene	*D. melanogaster*	*de novo*	Female reproduction	Lombardo et al. (2023)
Goddard protein	*D. melanogaster*	*de novo*	Sperm individualization	Lange et al. (2021)
Atlas	*D. melanogaster*	*de novo*	Spermatid chromatin condensation	Rivard et al. (2021)
555 *de novo* proteins	*D. melanogaster*	*de novo*	Mostly implied in fertility	Peng and Zhao (2024)
Tssor-3 and Tssor-4	*P. xylostella*	Orphan	Sperm count, fertility	Li et al. (2021)
lushu	*P. xylostella*	Orphan	Sperm maturation, motility	Zhao et al. (2024b)
60 *de novo* genes	Human	*de novo*	Mostly testis or cerebral cortex expression	Wu et al. (2011)
PBOV1	Human	*de novo*	Tumor-specific expression	Samusik et al. (2013)
6 *de novo* genes	Human	*de novo*	Testis-specific expression	Ruiz-Orera et al. (2015)
*De novo* lncRNA-derived genes	Human	*de novo* (debated)	Brain development (human-specific traits)	An et al. (2023)
Thousands of orphan genes	Human	Orphan	Tissue-specific regulation; potential disease links	Singh et al. (2024)
*de novo* ORF of SMIM45	Human	*de novo*	Brain development	Delihas (2024)
AFGPs	Codfishes (Gadidae)	*de novo* (debated)	Freeze protection	Baalsrud et al. (2018)
D6Ertd527e	Murid rodents	*de novo*	Oocyte expression	Petrzilek et al. (2022)
Dauerless	*P. pacificus*	Orphan	Dauer development	Mayer et al. (2015)
SELF-1	*P. pacificus*	Orphan	Self-recognition, cannibalism prevention	Lightfoot et al. (2019)
29 species-specific orphans	*P. pacificus*	Orphan	Niche adaptation, sperm-specific expression	Prabh and Rödelsperger (2019), Rödelsperger et al. (2021)
46 *de novo* genes	*C. elegans*	*de novo*	Involved in dauer stage and reproduction	Lee et al. (2022)

In examining various species, it is evident that the number of orphan genes and their representation among protein-coding genes varies significantly. In some species, such as *S. cerevisiae* and *Drosophila*, orphan genes can make up as much as 30% of the protein-coding genes. In contrast, this percentage is lower in species like the fungus *F. graminearum*, the nematodes *P. pacificus* and *C. elegans,* or humans, in which orphan genes comprise around 4% to 15% of protein-coding genes. These differences might reflect biological factors, including evolutionary pressures and unique genomic features of each species. However, these percentages are also highly dependent on the breadth and diversity of genomic data available, as well as methodological differences, and might be hardly comparable. Although most studies use similar approaches to identify orphan and *de novo* genes—homology search with comparative genomic tools, phylostratigraphy, alignment on closely related species and syntenic verification to classify *de novo* genes—the specific tools and parameters used can vary considerably. Different studies may apply different thresholds, scoring systems, and criteria leading to differing outcomes in orphan and *de novo* gene identification. Early studies in yeast and *Drosophila* primarily relied on straightforward but likely too simplistic BLAST homology searches with specific *e*-values against public databases. In contrast, more recent research increasingly incorporates comprehensive pipelines, employing advanced comparative genomic tools such as OrthoFinder (Emms and Kelly 2019), ORFan-Finder (Ekstrom and Yin 2016), OrthoMCL (Li et al. 2003), and HMMER (Finn et al. 2011) to systematically cluster and regroup homologous sequences. Therefore, differences can be observed even for the same species with different approaches. Automated standardized pipelines and file formats for the identification and description of orphan and de novo genes, such as the recently released DENSE (Roginski et al. 2024) and DeNoFo (Dohmen et al. 2025), will allow more comparable analysis at large scale in the future, providing these tools become largely used. Incorporation of ancestral sequence reconstruction in such pipelines is also expected to provide more precise identification of *de novo* genes in the future (Vakirlis et al. 2024). In addition to methodological differences, with time there were higher-quality annotated genomes available for more and more species, which explains the contradiction to orphan status of some genes in several species. Thus, the relative abundance of orphan genes within a species’ genome likely reflects not only inherent biological characteristics but also the diversity of methods and criteria used to identify orphan genes. Furthermore, it is also important to note that the orphan status varies between studies. Some studies focus on species-specific orphan genes, including species markers, while others focus on genus-specific ones. This highlights the need for caution when comparing orphan gene counts across studies, as variations in scope can impact results.

Another important consideration is the difference between highly divergent orphan genes and *de novo* genes. Most studies to date have suggested that only a small fraction of orphan genes arise *de novo*. However, in 2020, Vakirlis et al. provided important insights into the origin of orphan genes, challenging the assumption that high sequence divergence from ancestral genes is the primary cause of their orphan status (Vakirlis et al. 2020b). They re-analyzed orphan gene datasets from previous studies spanning multiple taxonomic groups, including yeast, flies, humans, and other vertebrates. Using a synteny-based pipeline developed in-house, they demonstrated that most orphan genes do not appear to have emerged by accumulating high divergence from pre-existing gene sequences, but rather from previously noncoding regions. Such findings highlighted the need for a revised perspective in orphan gene research, encouraging methodologies that are based on examining noncoding regions and transcriptional changes, rather than focusing solely on lack of homologs and sequence divergence. As a result, this study highlighted that *de novo* gene emergence may be more common than previously thought. However, it also suggested that there are limitations in using synteny to determine an ancestor due to genome rearrangements and other evolutionary events.

It should be noted that a study from 2024 suggested other hypotheses for the emergence of four of the *de novo* genes we describe in this review: BSC4 in yeast, Goddard in *Drosophila*, AFGP2 in codfish and FLJ33706 in humans (Hannon Bozorgmehr 2024). Based on remote homology relationships, this study suggested that these genes may have emerged through rearrangement and tinkering of previously-existing ones. However, this study relied on extremely relaxed BLAST parameters, yielding hits with high e-values and very low percent identity, within the range of the so-called twilight zone, casting doubts on the significance of homology. The other argument given was some structural similarity, but this does not necessarily imply inheritance from a common ancestral gene as it can equally be due to convergent evolution.

Anyhow, understanding the origin and mechanisms of emergence of orphan genes is still a difficult task. It depends on methods, genome and predicted proteome quality as well as all the criteria used.

Despite methodological challenges, the functional significance of orphan genes has been demonstrated across diverse species. Interestingly, in humans, insects, and nematodes, several orphans, including *de novo* genes, have been described to be specifically or particularly highly expressed in male gonads, with some having roles in spermatogenesis or reproduction (Table 1). The same observations have probably led Li Zhao and colleagues to wonder “Why are *de novo* genes predominantly enriched in the testis in animal species?” in the future issues section of their review on *de novo* genes (Zhao et al. 2024a). A possible explanation would lie in the pervasive transcription present in the testis, and particularly during late sperm maturation, with extensive chromatin remodeling facilitating the expression of many originally nongenic regions (Soumillon et al. 2013). This, associated with strong positive selection on testes, as an organ depending on sexual competition between males, might promote the emergence of new genes in that organ (Murat et al. 2023).

Besides reproduction, in humans, *de novo* genes such as SMIM45 (although later contradicted by Leushkin and Kaessmann 2024) and PBOV1 have been linked to cancer progression and brain development, respectively, highlighting their roles in physiological and disease contexts. In fungi, orphan genes like Osp24 in *F. graminearum* mediate host-pathogen interactions by modulating plant immune responses, while lineage-specific genes in ECM fungi are crucial for symbiosis with plant hosts. Similarly, in nematodes, orphan genes such as dauerless and SELF-1 regulate key survival strategies, including dauer development and self-recognition to prevent cannibalism. In codfishes, *de novo* antifreeze glycoproteins provide a survival advantage under freezing conditions, illustrating how environmental pressures can drive functional innovation. These examples demonstrate that orphan and *de novo* genes often evolve to fulfill specialized functions that address unique ecological, developmental, or reproductive challenges faced by their host organisms. This functional versatility underscores the significance of orphan genes as a rich source of evolutionary novelty, shaping specific traits and adaptations.

The study of orphan and *de novo* genes faces challenges; there's a need to define a reference methodology for accurate identification. Advances in sequencing technologies, computational tools, and experimental techniques offer solutions to these challenges. Integrating these approaches and fostering interdisciplinary collaboration can deepen our understanding of gene evolution and uncover applications in fields like biomedicine and agriculture.

This review has summarized the progress in understanding the prevalence, origins, and roles of orphan genes, particularly in well-studied model organisms like *Drosophila*, yeast, and humans but also in nonmodel organisms. Expanding research in nonmodel organisms highlights that these genes are neither rare nor insignificant in other lineages.

Moving on, paleogenomics will certainly offer a promising way to understand the origins of orphan and *de novo* genes. By comparing modern genomes with those of extinct species, we can identify ancestral homologs and distinguish true *de novo* emergence from cases of high divergence or gene loss. While its application is limited for now, advances in ancient DNA analysis could enhance our understanding of lineage-specific genes. Also, international projects like European Reference Genome Atlas and the Darwin Tree of Life are expected to greatly increase the number and diversity of high-quality genome assemblies. These efforts will improve comparative analyses across different groups of organisms and help us identify genes in previously underrepresented groups. Also, advances in environmental genomics and metagenomics can show us lineage-specific genes in uncultivated or cryptic organisms, helping us to understand more about gene emergence and diversity in natural populations.

As we look ahead, the study of orphan and *de novo* genes will undoubtedly continue to redefine our understanding of genomic and functional innovation (Xia et al. 2025), illuminating the remarkable capacity of life to generate novelty from previously considered “junk” genetic material. This knowledge holds the potential to address key scientific and societal challenges in the years ahead.

## Data Availability

This is a review paper and all the cited publications are publicly available. No new data has been generated here.
